# A Functional High-Load Exercise Intervention for the Patellar Tendon Reduces Tendon Pain Prevalence During a Competitive Season in Adolescent Handball Players

**DOI:** 10.3389/fphys.2021.626225

**Published:** 2021-03-10

**Authors:** Falk Mersmann, Gunnar Laube, Robert Marzilger, Sebastian Bohm, Arno Schroll, Adamantios Arampatzis

**Affiliations:** ^1^Department of Training and Movement Sciences, Humboldt-Universität zu Berlin, Berlin, Germany; ^2^Berlin School of Movement Science, Humboldt-Universität zu Berlin, Berlin, Germany

**Keywords:** tendinopathy, prevention, muscle-tendon imbalance, maturation, loading

## Abstract

Imbalances of muscle strength and tendon stiffness may increase the risk for patellar tendinopathy in growing athletes. The present study investigated if a functional high-load exercise intervention, designed to facilitate tendon adaptation and reduce muscle-tendon imbalances, may prevent patellar tendon pain in adolescent male handball players (12–14 years). Tendon pain prevalence (using VISA-P scores), knee extensor strength, vastus lateralis (VL) architecture and patellar tendon mechanical properties were measured at four measurement time points (M1–M4) over a season. The control group (CON; *n* = 18; age 13.1 ± 0.7 yrs, height 170 ± 8 cm, mass 58 ± 10 kg) followed the usual strength training plan, including muscular endurance and explosive strength components. In the experimental group (EXP; *n* = 16; 13.1 ± 0.6 yrs, 169 ± 11 cm, 58 ± 16 kg), two sessions per week with functional high-load exercises for the patellar tendon were integrated in the strength training schedule, aiming to provide repetitive high-intensity loading of at least 3 s loading duration per repetition. While in the control group 30% of the athletes reported a clinically significant aggravation of symptoms, all players in the experimental group remained or became pain-free at M2 until the end of the season. There was a similar increase of strength (normalized to body mass; CON: 3.1%, *d* = 0.22; EXP: 6.8%, *d* = 0.47; *p* = 0.04) and VL thickness (CON: 4.8%, *d* = 0.28; EXP: 5.7%, *d* = 0.32; *p* < 0.001) in both groups, but no significant changes of tendon stiffness or maximum tendon strain. Further, both groups demonstrated similar fluctuations of tendon strain over time. We conclude that functional high-load exercises can reduce the prevalence of patellar tendon pain in adolescent athletes even without a reduction of tendon strain.

## Introduction

In the production of movement, muscles and tendons work as a unit. While our muscles are able to generate forces and produce mechanical work, the elasticity of tendons enables the storage and release of strain energy, which in turn affects the operating conditions of the muscle with regard to its force-length-velocity relationship (Roberts, 2016; Bohm et al., 2019). However, muscle and tendon do not necessarily show a balanced adaptation to mechanical loading. Tendons have a lower rate of tissue turnover and adapt slower to increased loading compared to muscles (Kubo et al., 2010; Heinemeier et al., 2013). It also seems that there are differences in the mechanical stimuli which effectively trigger adaptation in these tissues. Plyometric loading for example shows clear effects on muscle size and strength (Sáez-Sáez de Villarreal et al., 2010), yet no or inconsistent effects on tendon stiffness (Fouré et al., 2009; Houghton et al., 2013; Bohm et al., 2014). An imbalanced adaptation of muscle strength and tendon stiffness during a training process reduces the tendon safety factor (i.e., ratio of ultimate to operating strain). This may lead to an increased risk of overuse injury considering the high prevalence of tendinopathy in sports featuring a high volume of plyometric loading due to jumps and change-of-direction movements (Lian et al., 2005; Mersmann et al., 2017a).

The prevalence of tendinopathy increases during adolescence (Simpson et al., 2016) and higher tendon strains due to muscle-tendon imbalances have been repeatedly observed in adolescent athletes compared to only recreationally active peers (Mersmann et al., 2016, 2017b, 2019; Charcharis et al., 2019). Further, it has been demonstrated in a group of adolescent basketball athletes that high levels of tendon strain are associated with a deterioration of the tendon’s structural appearance similar to tendinopathy (Mersmann et al., 2019). In that study, particularly high strains and low levels of structural organization of the tendon tissue were observed in athletes that already had or developed tendon pain in the following two months. These findings call for the development of interventions applicable to young athletes that complement the athletic training and strengthen their tendons, which might be of particular relevance in disciplines in which the risk of overuse tendinopathy is high and the sport-specific loading profile not optimal to trigger tendon adaptation (i.e., plyometric loading profile).

In a series of intervention studies (Arampatzis et al., 2007, 2010; Bohm et al., 2014), the factors that determine the mechanobiological response of tendons were systematically modulated (i.e., strain magnitude, rate, duration, and frequency) and it was possible to derive recommendations on how to train human tendons (Mersmann et al., 2017a). An effective training stimulus for the tendon is characterized by a repeated application of high-magnitude tendon strain (∼4.5–6.5%) over about 3 s. As the contraction type of the muscle does not directly affect tendon adaptation (Kjaer and Heinemeier, 2014; Bohm et al., 2015), this type of stimulus can be applied in a variety of isometric or dynamic exercises. For example, estimations of patellar tendon strain during single leg squats suggest a sufficiently high magnitude to trigger an anabolic response of the tendon (Kongsgaard et al., 2006). When performed slowly, the duration of target-range strain during squats should also be sufficient to be an effective stimulus for the tendon. Thus, it seems possible to use specific functional high-load exercises to facilitate tendon adaptation in athletes. The present study investigates the effects of a functional high-load exercise intervention for the patellar tendon, developed based on current scientific evidence on human tendon adaptation, applied to an adolescent group of handball athletes, which is a risk group for tendinopathy (Lian et al., 2005), over the course of a competitive season. A loading-induced increase of tendon stiffness would reduce tendon strain at a given force, which could prevent the development of structural impairments and pain in tendons (Backman et al., 2011; Mersmann et al., 2019). Therefore, we hypothesized that the intervention would reduce the prevalence of tendon pain and decrease imbalances between muscle strength and tendon stiffness compared to only sport-specific loading.

## Materials and Methods

### Participants and Experimental Design

**FIGURE 1:**
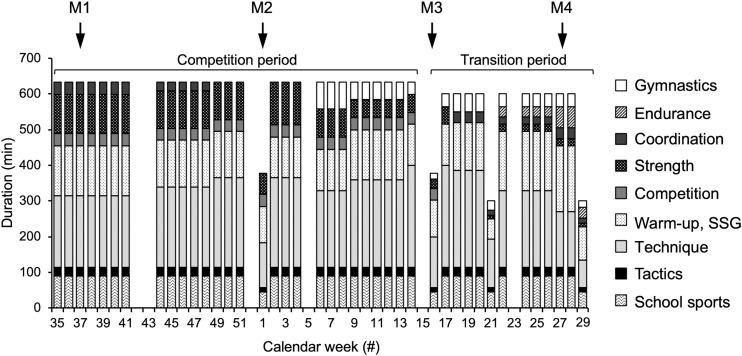
Total duration of respective training components of the adolescent handball athletes per calendar week. In the experimental group, the functional high-load exercise intervention was integrated into the strength training component, without a change of overall duration. Tendon pain, muscle strength and tendon mechanical properties were measured at the beginning (M1), during (M2) and at the end of the competition period (M3) and at the end of the transition period (M4) of the adolescent athletes’ season. SSG, small sided games.

In the present study, we aimed to examine patellar tendon pain prevalence as well as muscle and tendon properties of early-adolescent elite handball players at four measurement time points during a competitive season and the effect of the implementation of a functional high-load exercise intervention for the patellar tendon. The necessary sample size was calculated in a power analysis (G^∗^Power, version 3.1.6; HHU, Düsseldorf, Germany). We estimated a large effect (d = 1.2) of the intervention on the fluctuations of tendon strain (i.e., indicator for an imbalanced adaptation of muscle strength and tendon stiffness), considering the large differences we observed earlier between adolescent volleyball athletes and untrained peers (d = 1.8; Mersmann et al., 2016). For a power of 0.85, a sample size of 11 participants per group was calculated. Taking into account a potential drop-out, we recruited 18 top-level handball athletes in the age of 12–14 years from the seventh and eighth grade of a sports school associated to the Olympic Training Centre Berlin and assigned them to the control group (age 13.1 ± 0.7 yrs, height 170 ± 8 cm, mass 58 ± 10 kg), which received only their regular sport-specific training. In the following year, 16 athletes from the same grades (thus similar age) participated as experimental group (age 13.1 ± 0.6 yrs, height 169 ± 11 cm, mass 58 ± 16 kg), including five that had already been part of the control group. Figure 1 illustrates the weekly training duration and contents of the group, summing up—with a few exceptions—to at least 10 h in total, and the time points of the four measurements [M1: beginning of competition period (calendar week #37), M2: midway of competition period (calendar week #1), M3: beginning of transition period (calendar week #16) and M4: end of transition period (calendar week #27)]. Exclusion criteria were neurological or musculoskeletal impairments relevant for the purpose of the study, except patellar tendinopathy. The athletes were asked to fill out the validated German version of the VISA-P questionnaire (Lohrer and Nauck, 2011), considering the symptoms of the past week. With eight items, the instrument measures pain and functional impairments, including the self-perceived rating of pain on a ten-point Likert scale for selected every day and sport activities. A maximum score of 100 points reflects no pain and functional impairments. If the respective athlete confirmed that maximum strength testing was possible, the participant was included. The participants and their legal guardians gave written informed consent to the experimental procedures, which were approved by the Ethics Committee of the Charité, University Medicine Berlin (EA2/076/15), and carried out in accordance with the declaration of Helsinki. All measurements were performed on the dominant leg, which was defined as the leg used for kicking a ball. The biological maturity of the athletes was predicted using age and sitting height in the recalibrated prediction equation for boys suggested by Moore et al. (2015) and was at baseline −0.10 ± 0.61 and −0.09 ± 0.75 years to peak height velocity for the control and experimental group, respectively.

### Functional High-Load Exercise Intervention

The exercise program for the experimental group was designed with respect to earlier systematic research on human tendon adaptation *in vivo* (Arampatzis et al., 2007, 2010; Bohm et al., 2014). The most effective loading protocol for tendon adaptation from these studies was characterized by high-intensity loading for the tendon (to induce sufficient strain) for 3 s per repetition, applied in five sets of four repetitions with 3 s rest between repetitions and 2–3 min rest between sets. Taking this stimulus into consideration, we developed a collection of functional high-load exercises that (a) could be learned and performed by adolescents and (b) only involved equipment available in a regular gymnasium setting. The exercises were variations of squatting movements performed in single- or double-leg stance with or without additional loads. The eccentric-concentric movements were performed slowly and included an isometric phase of about 3 s at 90° knee joint angle to ensure a sufficient duration of high tendon loading. A list and illustrations of the exercises is available in the Supplementary Material of this article. We progressively increased the loading intensity over the season by advancing from body-weight exercises to using extra weights based on the skills and capacities of the athletes. The overall duration of the exercise program was about 20 min and was integrated twice a week in the strength training of the athletes. In the control group, the regular strength training consisted of explosive strength (low load, high velocity) and muscular endurance training (low load, high number of repetitions) and did not involve a training stimulus similar to the functional tendon exercises.

### Assessment of Vastus Lateralis Muscle Architecture

Vastus lateralis architecture was assessed at a knee joint angle of 60°, which has been reported earlier to be the approximate optimum knee angle for force production of the knee extensors (Herzog et al., 1990). A 10 cm linear ultrasound probe (MyLab 60; Esaote, Genova, Italy; probe: linear array (LA923), frequency: 7.5 MHz, depth: 7.4 cm, focal point: 0.9 and 1.9, no image filter) was placed over the belly of the inactive muscle in its longitudinal axis at ∼60% thigh length, which is the average location of the maximum anatomical cross-sectional area (Mersmann et al., 2015). The probe orientation was adjusted to enable the visualization of the longest possible fascicle fragments. The ultrasound images were analyzed offline using a custom written MATLAB interface (version R2016a; MathWorks, Natick, MA, United States). The upper and deeper aponeuroses were defined by setting reference points along the aponeuroses, which were then approximated by a linear least squares fitting. Subsequently, the visible features of multiple fascicles were identified using an automatic algorithm (Marzilger et al., 2018), which detects the boundaries of graphical objects (e.g., lines, circles) in a black-and-white converted image (for details see Marzilger et al., 2018). From the coordinates of the detected boundaries, the length and inclination of the fascicle fragments was calculated and weighted based on their length in the calculation of a representative reference fascicle (Marzilger et al., 2018). Fascicle length was calculated as the Euclidian distance between the intersection points of the reference fascicle with the two aponeuroses. The pennation angle refers to the angle between the reference fascicle and the deeper aponeurosis and muscle thickness to the average distance between the aponeuroses between the insertion points of the reference fascicle.

### Assessment of Quadriceps Muscle Strength

Muscle strength of the knee extensors was measured as maximum achieved knee joint moment (normalized to body mass) during maximum effort isometric voluntary contractions (iMVC) on a dynamometer (Biodex Medical System 3, Shirley, NY, United States). The resultant knee joint moments were calculated based on the inverse dynamics approach proposed by Arampatzis et al. (2004) which takes into account the inevitable axes misalignments of the knee joint and dynamometer during the course of the contraction as well as moments due to gravity. Normalized muscle strength was hence calculated as sum of the corrected maximum moment measured with the dynamometer (corrected using the ratio of the lever arms of the point of force application to the knee and dynamometer axis, respectively), the moment due to the mass of the shank-foot segment and the dynamometer lever arm, divided by body mass.

The necessary kinematic data were recorded using a Vicon motion capture system (version 1.7.1; Vicon Motion Systems, Oxford, United Kingdom) integrating eight cameras operating at 250 Hz. Five reflective markers were fixed to the following anatomical landmarks: greater trochanter, lateral and medial femoral epicondyles and malleoli, and two markers were fixed to the dynamometer: axis of the dynamometer and point of force application at the shank pad. Analog data were captured at 1,000 Hz and transmitted to the Vicon system via a 16-channel A/D converter. Both, the kinematic and analog data were low-pass filtered using a second-order Butterworth filter and a cut-off frequency of 6 Hz.

After a standardized warm-up, the participants were seated with a trunk angle of 85° (full hip extension: 0°) and the hip fixed to the dynamometer seat using a non-elastic belt. Ten submaximal isometric contractions with increasing effort served as additional warm-up, preconditioning of the tendon and familiarization. Subsequently, three maximum effort contractions were performed at resting knee joint angles of 65°, 70°, and 75° (full knee extension: 0°, values refer to the joint angle determined via the dynamometer), which were chosen based on our experience that participants reach their approximate optimum angle during contractions from these starting positions. Due to the non-rigidity of the human-dynamometer system (Arampatzis et al., 2004), the actual knee joint angles from the kinematic model corresponding to the maximum knee extension moments were on average 48 ± 8°. To account for the effect of gravity, an additional passive knee extension trial, driven by the dynamometer at 5°/s, was recorded with the shank of the participants fixed to the dynamometer lever pad.

### Measurement of Patellar Tendon Mechanical Properties

The force-elongation relationship of the patellar tendon was determined by combining inverse dynamics and ultrasound imaging. The probe of the ultrasound system was fixed in alignment with the longitudinal axis of the patellar tendon with a modified knee brace. The tendon elongation was captured during five trials of isometric ramp contractions (i.e., steadily increasing effort from rest to maximum in about 5 s). The knee joint angle was standardized for all participants to ∼60° at peak force (60.7 ± 3.3°). The knee joint moments were calculated as described above. To calculate patellar tendon forces, the joint moments were divided by the tendon moment arm, which was predicted based on anthropometric data (Mersmann et al., 2016) and adjusted to the respective knee joint angle based on the data reported by Herzog and Read (1993). The maximum tendon force (TF_max_) refers to the force calculated for the maximum iMVC trial of each participant. The elongation of the tendon during the ramp contractions was determined by tracking the displacement of the deep insertion of the tendon at the patella and tibial tuberosity in a custom-written MATLAB interface (version R2016a; MathWorks, Natick, MA, United States). Tendon slackness at rest was accounted for by measuring actual tendon elongation when the distance between the deep insertion points exceeded tendon rest length, which in turn was measured using a spline fit through the deep insertion marks and four additional points along the lower border of the slack tendon. The force-elongation relationship of the five trials of each participant was averaged to achieve an excellent reliability (Schulze et al., 2012). Tendon stiffness was calculated as slope of a linear regression between 50% TF_max_ and the highest common tendon force reached during the ramp contractions (which was on average 82 ± 7% TF_max_ and did not differ significantly between groups and measurement time points). Tendon strain is the maximum tendon elongation normalized to its rest length, which was on average 52.2 ± 5.4 mm.

### Statistics

Though five athletes participated in the control and experimental group, the groups were treated as independent samples in the statistical analysis, which is a more conservative approach compared to the assumption of dependent samples. Normality of the data was analyzed using the Shapiro-Wilk test. Baseline-data (M1) of age was compared between groups with a Student’s t-test for independent samples. All other parameters were analyzed with a linear mixed model for repeated measures and restricted maximum likelihood estimation using the nlme package in RStudio (version 1.2.1335, RStudio, Inc., Boston, MA, United States). Linear mixed models have the strength of being able to handle missing data and give the opportunity to take advantage of all information available for the statistical modeling in contrast with for example the ANOVA-models (Cnaan et al., 1997). Participants were included in the analysis if they took part in at least three of the four measurement time points, which applied to 15 and 13 participants in the control and experimental group, respectively. With regard to the tendon mechanical properties, the sample size reduced to 12 participants each, due to artifacts in the ultrasound imaging. In case of a significant interaction or main effect of time, *post hoc* tests were applied with Benjamini-Hochberg correction using the emmeans package of R, specifically testing differences between groups at M1 and differences between M1 and all other time-points analyzed separately for the control and experimental group. As these comparisons are based on the model estimates and not the descriptive means of the time points in question, they are not biased by missing data as simple t-tests. Cohen’s *d*, calculated based on the mean values predicted by the linear mixed model and the pooled standard deviation, will be reported for the comparison M1–M4 [with the subscript M1-4 for control (CON) and experimental group (EXP), respectively]. For tendon strain, we also compared the absolute residuals of the linear mixed model between groups (Mann-Whitney *U*-test) as a measure of imbalances in muscle and tendon adaptation over time (Mersmann et al., 2016). The VISA-P scores were analyzed with the linear mixed model as well, as they are robust against violations of the normality assumption. Further, we analyzed the frequencies of scores in 10-point-intervals as well as the frequencies of clinically relevant increases or decreases of the scores (± 13 points; Lohrer and Nauck, 2011; Hernandez-Sanchez et al., 2014). Three participants were excluded from the analysis of the VISA-P scores, due to knee joint pain unrelated to tendinopathy. The alpha level for all statistical tests was set to 0.05.

## Results

**FIGURE 2:**
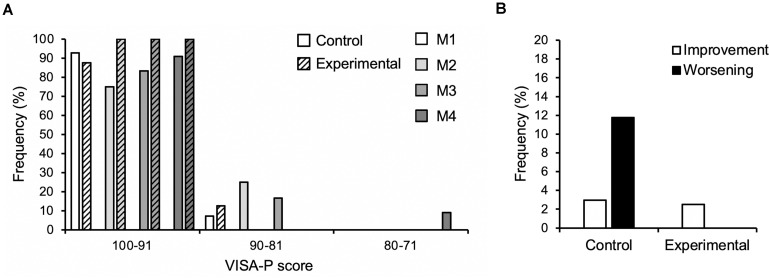
**(A)** Frequency of athletes reporting respective VISA-P scores in 10-point-intervals at the four measurement time points (M1-4) over the season in the control (*n* = 15) and experimental group (*n* = 16; hatched bars). **(B)** Frequency of clinically significant improvement or worsening (i.e., ±13 points of the VISA-P score) considering all observations in the control (*n* = 49) and experimental group (*n* = 56), respectively. Note that not all participants were able to attend all measurements.

**FIGURE 3:**
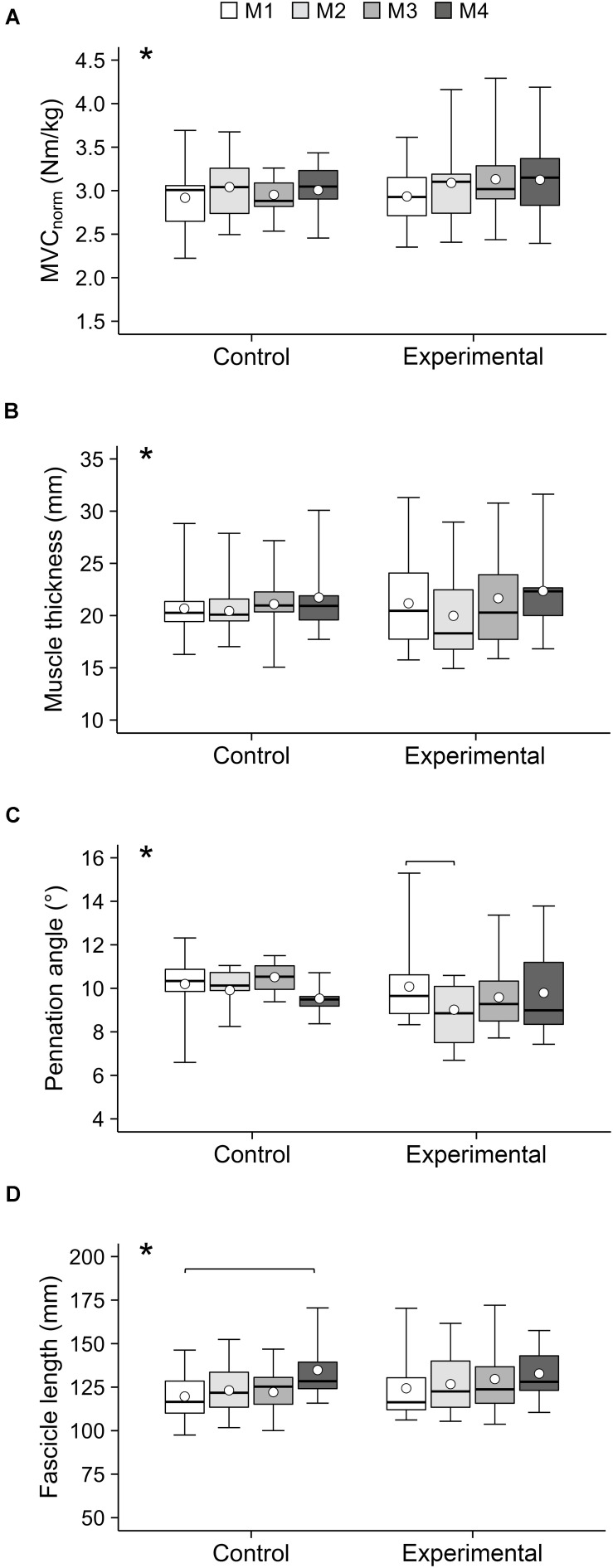
**(A)** Maximum isometric knee extension moment normalized to body mass (MVC_norm_), **(B)** vastus lateralis muscle thickness, **(C)** pennation angle and **(D)** fascicle length of the adolescent handball athletes in the control and experimental group at the four measurement time points (M1–M4) over the season. Note that not all participants were able to attend all measurements. The given experimental data are shown in box-plots, while the average values predicted by the linear mixed model are shown in white circles. *Significant main effect of time (*p* < 0.05). Significant *post hoc* comparisons are indicated by brackets (*p* < 0.05).

**FIGURE 4:**
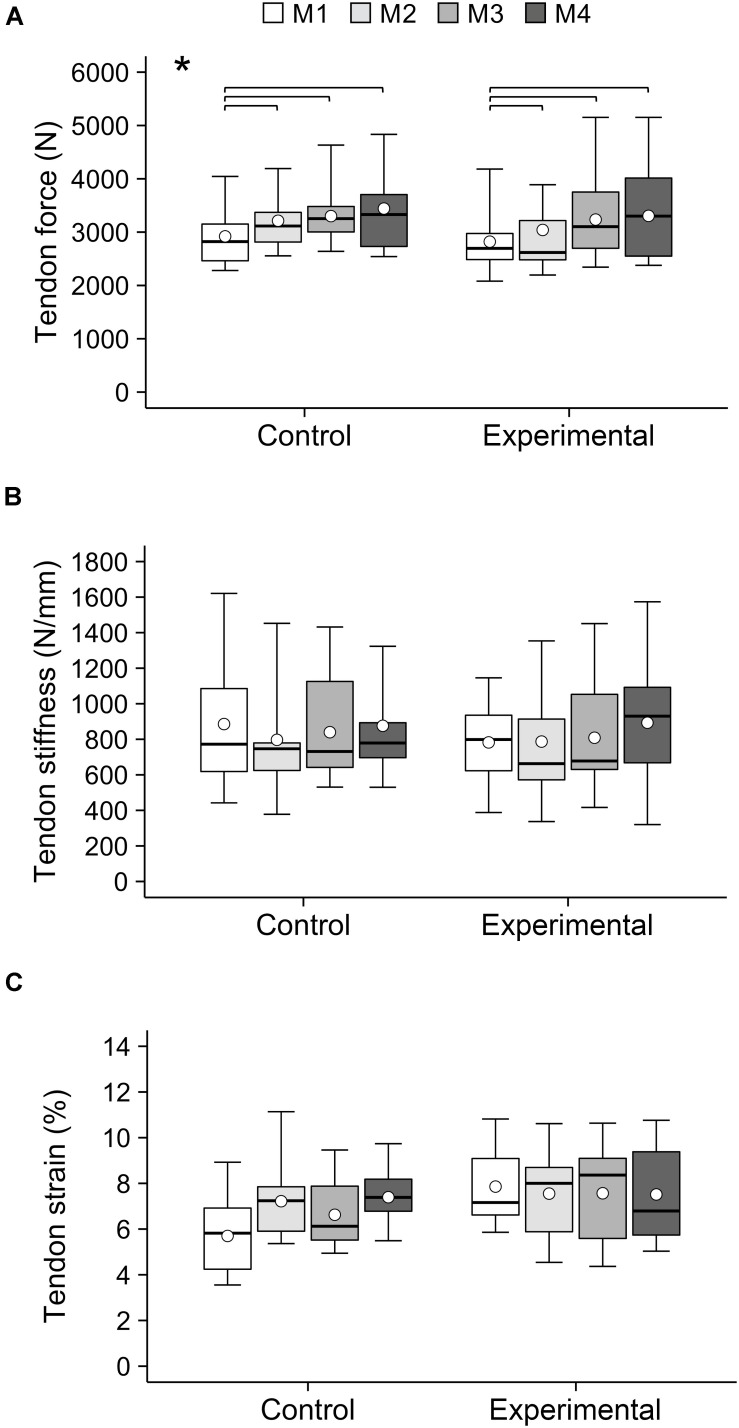
**(A)** Maximum tendon force, **(B)** stiffness and **(C)** strain of the patellar tendon of adolescent handball athletes in the control and experimental group at the four measurement time points (M1–M4) over the season. Note that not all participants were able to attend all measurements. The given experimental data are shown in box-plots, while the average values predicted by the linear mixed model are shown in white circles. *Significant main effect of time (*p* < 0.05). Significant *post hoc* comparisons are indicated by brackets (*p* < 0.05).

There was no significant difference in calendar age between groups at baseline (control: 13.1 ± 0.7 years, experimental: 13.1 ± 0.6; *p* = 0.92). There was a main effect of time on both body height and mass (*p* < 0.001; Table 1). There was a time-by-group interaction on body mass (*p* = 0.01), but, similar to body height, *post hoc* analysis showed a significant increase in body mass in both groups from baseline to all other measurement time points (*p* < 0.01) and no baseline difference between groups (*p* = 0.99). However, the effect sizes (body height: CON *d*_M__1__–__4_ = 0.63, EXP *d*_M__1__–__4_ = 0.5; body mass: CON *d*_M__1__–__4_ = 0.54, EXP *d*_M__1__–__4_ = 0.37) may suggest a more pronounced gain in body mass in the control group. Figure 2 shows the frequencies of the VISA-P scores in 10-point intervals as well as the rates of clinically significant worsening or improvement. The frequency of athletes reporting no relevant symptoms (i.e., 100–91 points) was quite similar at baseline (i.e., 93 and 88% in control and experimental group, respectively). While all athletes in the experimental group were pain-free from M2 to M4, the fraction of asymptomatic athletes decreased during the competition period and returned to baseline levels at the end of the transition period. Five out of 15 athletes in the control group (all of which had a VISA-score of ≥ 95 in the preceding assessment) but none of the 16 experimental group participants experienced a clinically significant worsening of the symptoms during the season, while one athlete reported an improvement of symptoms in both groups, respectively. The linear mixed model demonstrated a significant main effect of group on the VISA-P scores (*p* = 0.016) with lower average values in the control (M1: 95.6 ± 4.8, M2: 94.8 ± 6.5, M3: 96.5 ± 6.5, M4: 97.9 ± 6.6) compared to the experimental group (M1: 98.1 ± 4.1, M2: 99.9 ± 0.4, M3: 98.9 ± 2.4, M4; 99.3 ± 1.7). Time and interaction effects were not significant (*p* = 0.24 and 0.35, respectively). There was a significant main effect of time on body mass-normalized quadriceps muscle strength (*p* = 0.04), but no significant main effect of group (*p* = 0.50) or interaction (*p* = 0.44). However, in the *post hoc* analysis, none of the comparisons reached the level of significance (CON *d*_M__1__–__4_ = 0.22, EXP *d*_M__1__–__4_ = 0.49; Figure 3). Significant main effects of time were also observed on vastus lateralis muscle thickness (*p* < 0.001), pennation angle (*p* = 0.015) and fascicle length (*p* < 0.001). The data suggests an increase of muscle thickness (CON *d*_M__1__–__4_ = 0.28, EXP *d*_M__1__–__4_ = 0.32) and fascicle length (CON *d*_M__1__–__4_ = 0.91, *d*_M__1__–__4_ = 0.59) and a slight decrease of pennation angle; CON *d*_M__1__–__4_ = 0.5, EXP *d*_M__1__–__4_ = 0.21), yet *post hoc* comparisons were mostly non-significant (Figure 3). Tendon force increased significantly in both groups (time: *p* < 0.001, CON *d*_M__1__–__4_ = 0.77, EXP *d*_M__1__–__4_ = 0.72, group: *p* = 0.7, interaction: *p* = 0.85), with a significant change compared to baseline already at M2 (*p* < 0.05; Figure 4). However, there was no significant main effect of time (*p* = 0.75), group (*p* = 0.55) or time-by-group interaction (*p* = 0.78) on tendon stiffness (Figure 4). No significant main effects of time (*p* = 0.50) and group (*p* = 0.11) or time-by-group interaction (*p* = 0.07) was found for tendon strain (Figure 4) and the fluctuations of strain over time did not differ significantly between groups (CON 1.0 ± 0.5%; EXP 0.7 ± 0.3%; *p* = 0.38).

**TABLE 1:** Anthropometric data of the adolescent handball athletes in the control and experimental group for each measurement time point (M1-4) over a competitive season.

	Control *n* = 18				Intervention *n* = 16			
	
	M1	M2	M3	M4	M1	M2	M3	M4
	*n* = 17	*n* = 15	*n* = 15	*n* = 14	*n* = 16	*n* = 15	*n* = 13	*n* = 13
Maturity offset (years)*	−0.10 ± 0.61 (−0.10)	0.20 ± 0.64^‡^(0.23)	0.48 ± 0.64^‡^(0.48)	0.69 ± 0.67^‡^(0.73)	−0.09 ± 0.75 (−0.09)	0.24 ± 0.8^‡^(0.20)	0.44 ± 0.74^‡^(0.44)	0.64 ± 0.77^‡^(0.64)
Height (cm)*	170 ± 8 (170)	173 ± 10^‡^(173)	175 ± 9^‡^(175)	175 ± 9^‡^(176)	169 ± 11 (169)	171 ± 11^‡^(171)	172 ± 10^‡^(172)	173 ± 10^‡^(173)
Sitting height (cm)*	87.1 ± 4.5 (87.3)	88.8 ± 4.5^‡^(88.7)	89.6 ± 5.1^‡^(89.6)	90.0 ± 4.9^‡^(90.5)	87.2 ± 6.5 (87.0)	88.5 ± 6.6^‡^(88.1)	89.1 ± 5.4^‡^(89.1)	89.9 ± 5.5^‡^(89.9)
Mass (kg)*	58.2 ± 10.4 (58.6)	62.9 ± 9.7^‡^(61.3)	64.3 ± 11^‡^(64.3)	65.1 ± 11^‡^(65.9)	57.8 ± 15.5 (58.6)	59.5 ± 15.8^‡^(60.2)	62.2 ± 15.4^‡^(62.2)	63.6 ± 16.1^‡^(63.6)

Note that not all participants were able to attend all measurements. The numbers are average ± SD of the given experimental data. The values in brackets show the group average predicted by the linear mixed model. Maturity offset refers to the estimated years to peak height velocity. *Significant main effect of time (p < 0.05); ^‡^significantly different to M1 (post hoc test; p < 0.05).

## Discussion

The present study investigated the effects of a functional high-load exercise intervention for the patellar tendon on pain prevalence, knee extensor muscle strength and vastus lateralis architecture as well as patellar tendon mechanical properties in adolescent handball players, assessed at four measurement time points over a competitive season. In comparison to a control group that followed the regular training schedule, patellar tendon pain frequency and the rate of clinically significant aggravations of symptoms was lower in the athletes that received the intervention. However, the data did not substantiate the expected benefits considering imbalances of muscle strength and tendon stiffness, as tendon strain and strain fluctuations over the whole year were similar between groups. Therefore, our hypotheses were only partly confirmed.

The functional exercise intervention applied in the current study was designed based on current knowledge on the properties of an effective training stimulus for tendon adaptation (Arampatzis et al., 2007, 2010; Bohm et al., 2014) and promising results were achieved with regard to the prevention of patellar tendon pain in adolescent handball athletes. While 30% of the athletes in the control group (∼12% of the observations) experienced a clinically significant worsening of tendon pain during the season, all of the intervention participants were pain-free from the second measurement session until the end of the season. The effectiveness of the applied intervention for the reduction of pain prevalence has important implications considering that tendinopathy is a major issue in many popular sports (Lian et al., 2005). More than every second athlete with patellar tendinopathy reports a reduction of sport performance due to pain (De Vries et al., 2017), which may imply a profound decrease in quality of life when physical capabilities are a crucial part of an individual’s identity (Ceravolo et al., 2018). Although the prevalence of tendinopathy in the early adolescence is lower compared to late adolescence and adulthood in athletes (Simpson et al., 2016), the time period around the peak height velocity is very sensitive in terms of appropriate mechanical loading for a successful athletic career (Lloyd et al., 2015). Particularly, plyometric loading (either as training modality or due to sport-specific training) may put young athletes at risk for tendon overuse, considering the loading-profile of high-risk sports for tendinopathy and the dissimilar effects on muscle and tendon adaptation (for review see Mersmann et al., 2017a). Thus, implementing the functional high-load exercise intervention for the tendon into models of physical development of youth athletes may have a relevant contribution for injury prevention when the volume of plyometric loading increases during adolescence.

Against our expectations, we did not observe an effect of the intervention on the prevalence of musculotendinous imbalances. Fluctuations of tendon strain during maximum muscle contractions serve as an indicator of imbalances in muscle and tendon adaptation over time, which can cause periods during an athletic training in which the tendon is subjected to potentially detrimental levels of tendon strain (Mersmann et al., 2016). These fluctuations were similar in both groups (control 1.0%, intervention 0.7%) and comparable with the increased fluctuations that were found in mid-adolescent volleyball athletes (0.9%) in comparison to untrained peers (0.3%; Mersmann et al., 2016). Therefore, it seems that the benefits observed considering tendon pain prevalence were not due to a more uniform muscle and tendon adaptation and prevention of high-level tendon strain.

Though strain-induce overload is considered a potential key factor in the etiology of tendinopathy (Arnoczky et al., 2007; Magnusson et al., 2010; Cook et al., 2016), the magnitude of habitual tendon strain is unlikely to explain the reduction of pain prevalence in the experimental group. Recent work by Tran et al. (2019) suggests that early-stage tendinopathy is characterized by the expression of nociceptive mediators and a disturbed tissue homeostasis. Therefore, it could be speculated that the type of loading applied in the experimental group may have induced beneficial metabolic effects for the maintenance of the biochemical milieu (Khan et al., 2000). Considering the changes in mechanical properties observed in our earlier intervention studies (Arampatzis et al., 2010; Bohm et al., 2014), it seems that the duration of tendon strain during loading cycles has an important influence on the metabolic response to loading. A longer load-application—as emphasized in the exercises for the experimental group—may lead to a more homogeneous strain-distribution within the extracellular matrix due to the viscoelastic properties of the tissue (Baar, 2019) and more uniform stimulation of the embedded tenocytes compared to the sport-specific plyometric loading. Though the type of loading applied in the experimental group may not have led to a net anabolic metabolism and change of the tendon mechanical properties, it could still have been beneficial for maintaining tissue homeostasis and reducing nociceptive signaling.

Though the athletes performed the functional high-load exercise intervention for almost a year and tendon adaptation should be possible to achieve in early adolescence (Charcharis et al., 2019), we did not observe a significant increase of tendon stiffness and reduction of tendon strain in the experimental group. Due to the functional nature of the exercises, we were not able to control the actual strain of the tendon during the training. Yet, at least in the highest loading conditions (i.e., single-leg squats with additional weights), which were applied over 16 weeks, the joint moments and tendon forces should have been of sufficient magnitude (Zwerver et al., 2007) to reach appropriate levels of tendon strain to trigger tendon adaptation (i.e., 4.5–6.5%; Arampatzis et al., 2007; Wang et al., 2013). However, in the earlier intervention studies, on which the functional high-load exercise program was based on, four training sessions per week were used to stimulate tendon adaptation (Arampatzis et al., 2007, 2010; Bohm et al., 2014). Thus, two sessions of functional high-load exercises per week might not have been sufficient to elicit a change in tendon mechanical properties considering the low loading volume of a single tendon-specific training session. Another factor that could have reduced the efficacy of the training might be that the relationship of loading intensity and tendon strain is quite individual. This means that when two athletes train with the same intensity (either absolute or relative to maximum strength), inter-individual differences in tendon stiffness may cause substantial deviations in the mechanical demand for the tendon, especially in groups that are predisposed for imbalances of muscle strength and tendon stiffness (Arampatzis et al., 2020). The high variability of tendon strain during maximum muscle contractions and in the change of tendon stiffness during the season (19 ± 56% in the experimental group) provide some support for this assumption.

With regard to muscular properties, a significant effect of time was observed on knee extensor muscle strength and vastus lateralis thickness and the lack of time-by-group interactions suggests that these changes were similar in both groups. As both an increase of muscle strength and size can be achieved with a wide range of loading modalities and the overall duration of strength training was the same in both groups, this was not surprising. Due to the lack of an untrained control group, it remains unclear to what extent the change in muscle strength and thickness were due to maturation and training. Yet, it may still be an important message that the proposed program for the prevention of tendon pain can be implemented in a training schedule for young elite athletes without compromising the development of muscle strength and hypertrophy.

Without any time-by-group interaction, there was an increase in fascicle length and a reduction of pennation angle (i.e., significant main effect of time). However, in the *post hoc* tests the increase of fascicle length was only significant in the control group between M1 and M4. This may be related to the on average greater increase in body height in the control group (6 cm vs. 4 cm in the intervention group). The decrease in pennation angle was unexpected, considering that earlier reports rather suggest an increase of pennation angle in response to athletic training, even in early adolescence (Mersmann et al., 2020). However, the rapid longitudinal growth of the muscle during the adolescent growth spurt may induce opposing effects. We recently found that the vastus lateralis muscle shape (in terms of the ratio of average to maximum anatomical CSA) is different in growing adolescents compared to adults, which may point towards a growth-related remodeling of the muscle. The transient significant change of vastus lateralis pennation angle between M1 and M2 in the intervention group may be a combination of the pronounced growth in this period and the potentially lower loading intensity in the beginning of the intervention, when the athletes in the intervention group were familiarized with the target exercises while the controls were able to continue their habitual strength training routines.

There are some limitations to this study that warrant discussion. A common issue of studies on adolescents is the lack of adequate control of maturity status without invasive measures. Though anthropometric-based predictions offer a practical approach to estimate maturity, uncertainties about the actual biological age remain. Similarly, the actual loading both during the common training and exercises introduced in the experimental group cannot be controlled in a study that aims to translate knowledge from fundamental science (in terms of effective loading for tendons) into the practical field using functional high-load exercises instead of laboratory-controlled loading. Future studies may investigate the actual tendon loading during such exercises. Finally, due to the time-consuming procedures involved with the assessment of tendon mechanical properties and muscle architecture *in vivo*, the sample size in this study was rather small with regard to the outcome of pain and may need support from future epidemiological studies. Nevertheless, this study presents unique longitudinal data from a heavily under-investigated cohort, measured using a state-of-the-art experimental approach for the assessment of tendon mechanical properties *in vivo* (Seynnes et al., 2015) and provides promising results for the prevention of tendon pain. Incorporating five sets of four slow repetitions of high-intensity exercises (to induce sufficient tendon strain for at least 3 s) twice a week into the athletic training in sport disciplines associated with predominantly plyometric loading and a high risk of tendinopathy may significantly contribute to reduce the prevalence of tendon pain in young athletes.

In conclusion, the present study demonstrated that a functional exercise intervention for the tendon, which—based on our current knowledge on tendon adaptation—aims to provide repetitive long-duration, high-intensity loading and does not require special equipment, can reduce the prevalence of patellar tendon pain in adolescent athletes. However, the positive effect could not be attributed to the prevention of imbalances of muscle strength and tendon stiffness and reduction of tendon strain. In its current form, the functional tendon exercise program may be recommended to complement the athletic training in risk groups for tendinopathy to achieve positive outcomes on the risk of tendon pain without a compromise of muscle strength development.

## Data Availability Statement

The raw data supporting the conclusions of this article will be made available by the authors, without undue reservation.

## Ethics Statement

The studies involving human participants were reviewed and approved by the Ethics Committee of the Charité, University Medicine Berlin (EA2/076/15). Written informed consent to participate in this study was provided by the participants’ legal guardian/next of kin. Written informed consent was obtained from the individual(s) for the publication of any potentially identifiable images or data included in this article.

## Author Contributions

FM and AA conceived the experiment, interpreted the data and drafted the manuscript. GL and RM performed the experiments. FM and GL analyzed the data. RM, SB, AS, and AA substantially contributed to data analysis. GL, RM, SB, and AS made important intellectual contributions during revision. All authors approved the final version of the manuscript and agreed to be accountable for the content of the work.

## Conflict of Interest

The authors declare that the research was conducted in the absence of any commercial or financial relationships that could be construed as a potential conflict of interest.
