# Supplementary material for: A Functional High-Load Exercise Intervention for the Patellar Tendon Reduces Tendon Pain Prevalence During a Competitive Season in Adolescent Handball Players
Source: Front Physiol. 2021 Mar 10;12:626225. doi: 10.3389/fphys.2021.626225 (PMC7987778; doi:10.3389/fphys.2021.626225)
Supplement: Supplementary file 1 [file Data_Sheet_1.PDF]

Supplementary Material

1 Tendon training

Loading characteristics for all exercises:

- Frequency: 2 times per week
- Number of sets: 5
- Repetitions per set: 4
- Intensity: moderate to high
- Loading duration per repetition: 3-6 s
- Rest between sets: 1-2 min
- Duration of the entire training session: about 15 min

CW: Calendar week

CW 41 – 44: 4 weeks including 2 weeks holidays with home-based training

Single-leg squats

Starting position is a one-leg stand with one hand on the wall (for balance support) and the other leg stretched out in front. The supporting leg is slowly bent to 90° knee angle, the position is held isometrically for 3 s. The supporting leg is extended to return to the starting position. After 4 repetitions legs are switched.

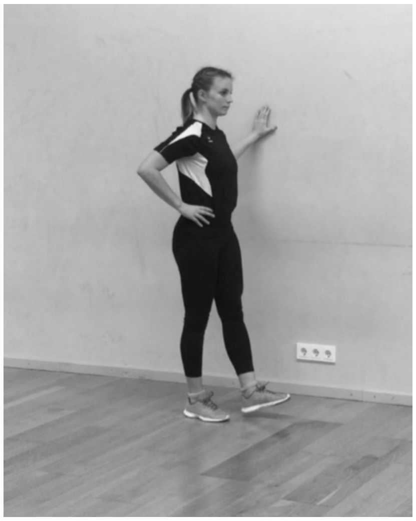

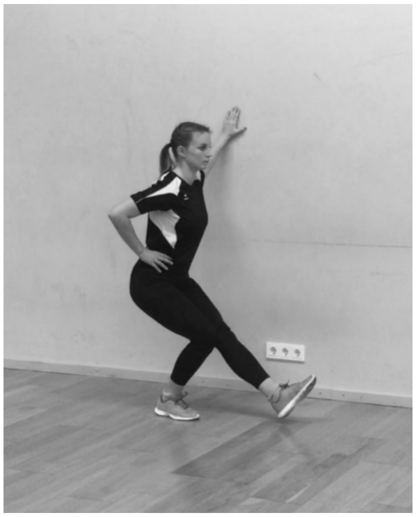

CW 45 – 47: 3 weeks

Single-leg stepping on bench

Starting position is one leg on a bench, the other leg behind it on the floor. The weight is shifted to the front leg, which is then slightly extended (to a position of about 90°) until the back leg is lifted off the floor. The position is held isometrically for 3 s, then the back leg is lowered back down on the floor. After 4 repetitions legs are switched.

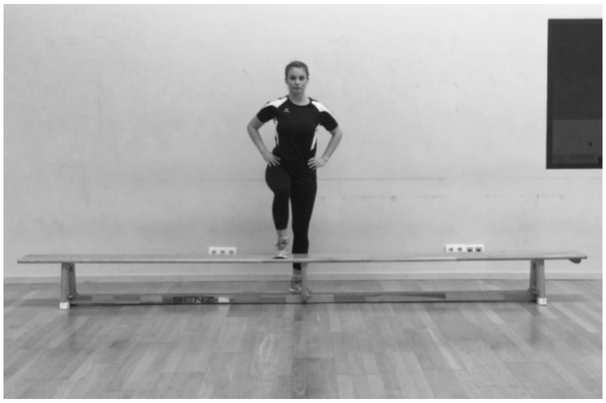
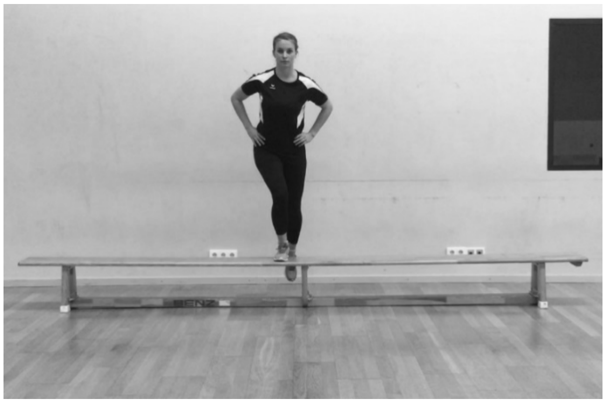

CW 48 – 2: 7 weeks including 2 weeks holidays

Single-leg squats with bench

To add extra weight, two athletes hold a bench between them at chest-height while performing single-leg squats.

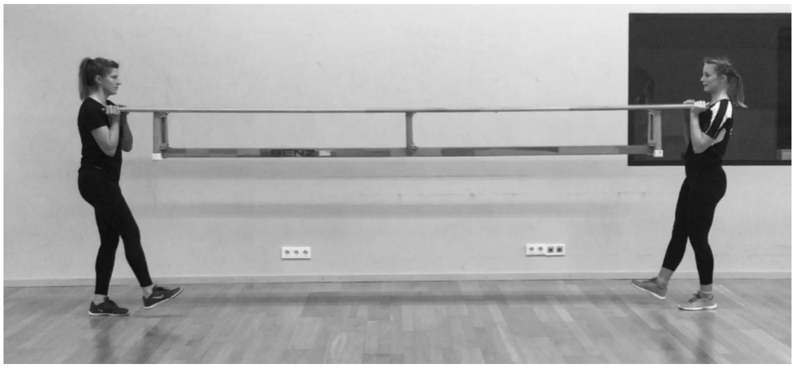
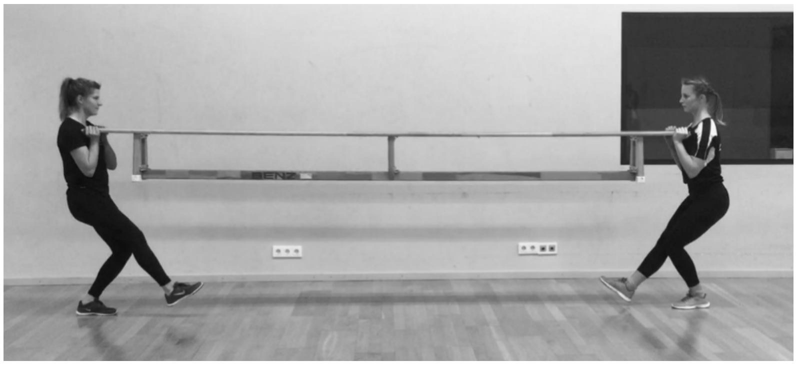

CW 3 – 8: 6 weeks including 1 week holiday

Squats with partner piggyback

Starting position is a stand with the feet hip-width apart while carrying a partner piggyback. Both legs are slowly bent to 90° knee angle. The position is held isometrically for 3 s, then the legs are extended to return to the starting position. After 4 repetitions partners switch.

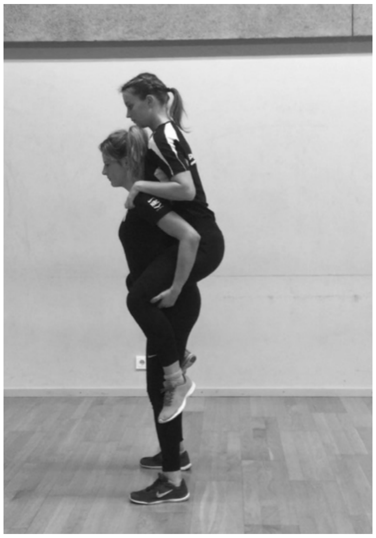

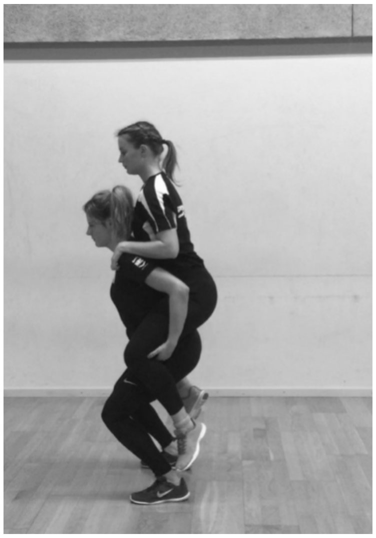

CW 9 – 16: 8 weeks including 2 weeks holidays

Single-leg squats with partner piggyback

Starting position is a stand with the feet hip-width apart while carrying a partner piggyback. Both legs are slowly bent to 90° knee angle. The body weight is shifted to one leg, the other leg is lifted off the floor. The position is held isometrically for 3 s, then the free leg is lowered back down on the floor and both legs are extended to return to the starting position. After 4 repetitions partners switch, for the next set legs are switched. Another team of two athletes provides support (not shown here).

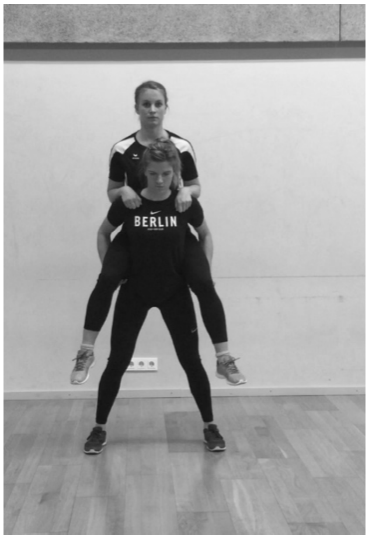

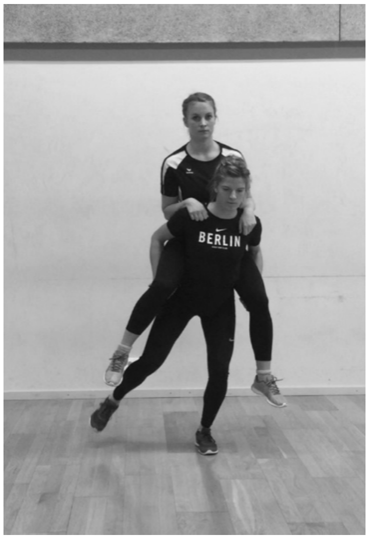

CW 17 – 24: 8 weeks

Variation of single-leg squats with partner piggyback

Start like single-leg squats with partner piggyback. In the bent position (about 90° knee angle) first one leg is lifted off the floor and the position is held for 3 s, then the other leg is lifted off the floor and the position is held for 3 s. Then, the free leg is lowered back down on the floor and both legs are extended to return to the starting position. After 4 repetitions partners switch. Another team of two athletes provides support.

Written informed consent was obtained from the individuals shown for the publication of these images.
